# Prevalence and characteristics of epilepsy in the Belgian shepherd variants Groenendael and Tervueren born in Denmark 1995–2004

**DOI:** 10.1186/1751-0147-50-51

**Published:** 2008-12-22

**Authors:** Mette Berendt, Christina Hedal Gulløv, Stine Louise Krogh Christensen, Hulda Gudmundsdottir, Hanne Gredal, Merete Fredholm, Lis Alban

**Affiliations:** 1 Department of Small Animal Clinical Sciences, Faculty of Life Sciences, University of Copenhagen, Dyrlægevej 16, DK-1870 Frb. C, Denmark; 2 Department of Basic Animal and Veterinary Sciences, Faculty of Life Sciences, University of Copenhagen, Grønnegårdsvej 7, DK-1870 Frb. C, Denmark; 3 Danish Meat Association, Axelborg 3, DK-1609 Copenhagen K, Denmark

## Abstract

**Background:**

The Belgian shepherd Groenendael and Tervueren is believed to be at higher risk of developing epilepsy than dogs of the common population. This epidemiological study was designed to estimate the prevalence of epilepsy in the Danish population of Groenendael and Tervueren born between 1995 and 2004. Furthermore, it was the intention to describe the clinical manifestation (seizure types and phenomenology) of epilepsy and to identify risk factors for euthanasia once the dog was diagnosed as having epilepsy.

**Methods:**

All owners of Groenendael and Tervueren dogs born between January 1995 and December 2004 and registered in the Danish Kennel Club (1,248 dogs) were contacted and asked to answer a mailed questionnaire concerning epilepsy. Positive responders were subsequently validated in a follow-up interview conducted by telephone using a standardized questionnaire. Owners were questioned about age at first seizure, seizure frequency, seizure duration, a detailed description of seizure phenomenology, post-ictal signs and if a veterinarian had diagnosed the dog with epilepsy.

**Results:**

Prevalence of epilepsy was estimated at 9.5%. Mean age of epilepsy debut was 3.3 years (range 0.5–8.0 years). There was an almost equal number of Groenendael (25) and Tervueren (24). The distribution of females and males was 31 and 18 respectively. Twenty-five per cent experienced focal seizures, 53% experienced focal seizures with secondary generalization and 18% experienced primary generalized seizures. In four percent seizures were unclassifiable. The most commonly reported focal seizure phenomenology included ataxia, crawling, swaying, fearful behavior, salivation, excessive attention seeking and disorientation. In 16% of the cases, epilepsy led to euthanasia. Intact dogs with epilepsy had a significantly increased risk of being euthanized because of epilepsy compared to neutered dogs with epilepsy. In 22% of the cases the owners reported that anxiety/hyperactivity/stress could act as a seizure provoking factor.

**Conclusion:**

A high prevalence of epilepsy appears to be present in the Danish Groenendael and Tervueren population. The relatively late debut age of epilepsy in this breed contributes greatly to the increased prevalence of epileptic individuals, because dogs developing epilepsy late in life are used for breeding unintended.

## Introduction

Epilepsy is a neurological condition characterized by episodic cerebral neuronal dysfunction leading to seizures with convulsion or more focal signs as, i.e. localized motor signs, autonomic signs or paroxysms of behavioral signs (corresponding to a response provoked by alterations in cerebral neuronal activity in special sensory areas – in humans referred to as psychic signs). The severity of the disease is reflected in the increased risk of premature death, due to i.e., therapeutic failure or/and the amount of emotional strain on the owner of the epileptic animal [1].

Genetic factors are increasingly being identified as the underlying mechanisms of specific types of epilepsy in both humans and canines [2-4]. In dogs, some breeds express an accumulation of epileptic individuals in the population. Among others, the Belgian shepherd, Beagle, Keeshound, Labrador retriever, Bernese mountaindog, Shetland sheep dog, Irish wolfhound, English springer spaniel and Lagotto Romagnolo, have shown an increased risk of epilepsy with a suspected genetic background [5-15].

Almost 40 years ago, it was suggested that epilepsy in the Belgian Tervueren could be inherited [16]. The heritability in this breed in the USA has been estimated at 0.77 [7,17]. Pedigree analyses have rejected a Mendelian inheritance. However, it has been suggested that a single gene with a large impact is implicated [7,12,17,18].

The Belgian shepherd consists of four variants: Groenendael, Tervueren, Malinois and Laikenois. The Groenendael variant was introduced in Denmark in 1968 by import of a Swedish Groenendael bitch. Dogs from this breeding line produced almost 500 puppies during the following 10 years, and in the early 1980's almost 95% of the Groenendaels in Denmark descended from this line. From the mid 1980's many Groenendaels were imported from especially the Netherlands, Switzerland, Belgium and France and new breeding lines were hence created. The first Danish litter of the Tervueren variant was born in 1978.

In recent years, breeders of Groenendael and Tervueren in Denmark have reported an unusual increase in the number of individuals with epilepsy.

The aim of the study was to estimate the prevalence of epilepsy in the Danish Groenendael and Tervueren population. Additionally, we wanted to describe the characteristics of epilepsy, including seizure phenomenology and the distribution of seizure types in the breed. Finally, we wanted to identify risk factors for euthanasia once a dog was diagnosed with epilepsy.

## Methods

The study was carried out in 2005. The prevalence study was designed as a retrospective population study. The reference population consisted of all Groenendael and Tervueren dogs born between January 1^st ^1995 and December 31^st ^2004 and registered in the central register of the Danish Kennel Club (DKC), a total of 1,248 dogs. The DKC register contains information about all Danish purebred dogs. Dogs are listed chronologically by date of registration, and Danish purebred dogs must be registered before the age of three weeks.

The study was conducted in three steps (I-III).

### Step I – Initial information campaign regarding the study

Breeders and owners of Groenendael and Tervueren were informed about the forthcoming prevalence investigation of epilepsy in the breed through the DCK and the journal and homepage of the Belgian Shepherd Club.

### Step II – mailed questionnaire survey

A questionnaire was posted by the DKC to the owners of the 1,248 dogs comprising the reference population. The questionnaire included a short introduction to epilepsy, followed by a list of typical clinical signs seen in focal seizures, focal seizures with secondary generalization and primary generalized seizures. In the description of epilepsy, it was emphasized that epileptic seizures are of short duration (seconds to minutes), and that the seizure phenomenology will be identical from seizure to seizure within the individual. The owners were asked to mark if their dogs had shown any of the signs of epilepsy listed. If the dog had died, the owners were asked to state the year of death and cause of death. Finally, the owners were asked to give a written consent to allow the investigators to contact them by telephone.

### Step III – Validation of positive responders

Returned questionnaires were evaluated by the investigators (MB, SLKC, HGu) and divided into two groups: negative responders and possibly positive responders (self-reported epilepsy). Subsequently, all possibly positive responders were contacted by telephone and interviewed in order to validate if the clinical signs and seizure history experienced by the individual dog were in agreement with those of a typical epileptic seizure phenomenology and seizure history. The interviewers used a validated questionnaire containing questions regarding age at first seizure, seizure frequency, seizure duration, as well as a detailed description of seizure phenomenology and post-ictal signs. Owners were also asked if the dog had been diagnosed with epilepsy by a veterinarian and about antiepileptic medication. Finally, the owners were asked if the dog was known to suffer from diseases other than epilepsy and if the dog appeared normal interictally. If the clinical signs described by the owners could be mistaken for a possible differential diagnosis to epilepsy, clarifying questions were asked in order to rule out diseases that might mimic epileptic seizures. A typical telephone interview lasted 30–45 minutes.

### Case definition

The diagnosis of epilepsy was based upon the detailed information collected on seizure history, seizure phenomenology and development, duration and characteristics of the disease, as also used in humans [19].

In this study a dog was defined as an epilepsy positive case if:

1) The information collected from the owner of a possible epilepsy positive dog (identified in step 1) in the telephone follow-up interview could be confirmed to comply with the criteria of a typical description of epileptic seizure phenomenology and the typical characteristics and history of epilepsy

and

2) the dog had experienced two or more seizures (as defined by the Commission on Epidemiology and Prognosis, ILAE [20]).

### Risk factors

Associations between cause of death and sex, cause of death and variant (Groenendael/Tervueren), type of seizure and variant (Groenendael/Tervueren), and neutering status and cause of death were investigated by use of Fisher's exact test.

### Miscellaneous

Pedigrees were collected on all dogs included in the study.

Additional information was collected from owners of dogs identified by breeders as epilepsy suspect, but where the owners did not return the mailed questionnaire. These dogs were investigated using the methods described in step III.

## Results

### Study population and prevalence

A total of 1,248 Groenendael and Tervueren dogs were included in the study. Out of the 1,248 mailed questionnaires, 526 were returned. Seven responders were excluded because the questionnaires were incompletely filled in, leaving 519 questionnaires to be evaluated (corresponding to a study population of 519 individuals). Hence, the return rate of the mailed questionnaire was calculated to 42%.

Out of the 519 responders, 64 owners (12.3%) responded positively to the questions about epilepsy (self reported epilepsy) while the remaining 455 (87.7%) responded negatively.

At the time of the study, a total of 79 dogs from the overall study population of 519 dogs had been euthanized. The most common cause of euthanasia was aggression (27 dogs), cancer (14 dogs), epilepsy (eight dogs), high age (seven dogs) and traffic accidents (four dogs).

Out of the 64 possibly epilepsy positive responders that were listed to be telephone interviewed, three owners could not be reached and were therefore excluded. After evaluating the results of the 61 telephone interviews, 49 dogs were finally confirmed to fulfill the criteria of being epilepsy-positive cases; 25 Groenendael (15 females and ten males) and 24 Tervueren (16 females and eight males). Hence, there was an overrepresentation of females (31) with epilepsy compared to males (18). Thirteen dogs were neutered (nine females and four males) (Table 1).

**Table 1:** Distribution of factors related to 49 DanishGroenendael and Tervueren dogs diagnosed as epilepsy-positive in a study including 519 dogs.

**Factor**	**No. (%)**
Sex	
*Female*	31 (63.3)
*Male*	18 (36.7)
Variant	
*Groenendael*	25 (51.0)
*Tervueren*	24 (49.0)
Diagnosed by practicing veterinarian	
*Yes*	35 (71.4)
*No*	14 (28.6)
Type of seizure	
*Focal seizures alone*	12 (24.5)
*Focal seizure with sec. generalization*	26 (53.1)
*Primary generalized seizures*	9 (18.4)
*Unclassifiable seizures*	2 (4.1)
Cause of death^1^	
Epilepsy	8 (53.3)
*Other causes*	7 (46.7)
Neutering status^2^	
Neutered	13 (27.1)
*Intact*	35 (72.9)

^1^Only calculated for the 15 dogs that had died.

^2^One missing value for this variable.

The prevalence of epilepsy was estimated at 9.5% (49/516). A preliminary interpretation of pedigrees disclosed that a familiar connection existed among several of the affected dogs.

### Dogs identified by breeders/owners

Breeders reported that several owners of dogs known to them had not returned the mailed questionnaire despite having dogs diagnosed with epilepsy by a veterinarian. This information yielded the identities of an additional ten dogs with epilepsy. The owners were contacted and interviewed using the standardized questionnaire also used in the telephone interview (step III). All ten dogs fulfilled the criteria of being epilepsy positive according to the study case definition. The prevalence of epilepsy would have been higher if these dogs had been included. However, since these owners did not return the mailed questionnaire, they did not fulfill the study design criteria. Hence, the extra ten cases could not be included in the calculation of prevalence.

### Risk factors

Associations between cause of death and sex, cause of death and variant (Groenendael/Tervueren), type of seizure and variant (Groenendael/Tervueren), and neutering status and cause of death were investigated (Table 1). Only the last association was found to be statistically significant. Intact dogs (not-neutered) with epilepsy had an increased risk of being euthanized because of epilepsy compared to neutered dogs with epilepsy (N = 15, Fisher's exact test *P *= 0.007). Neither the relative risk (RR), nor the odds ratio (OR) could be calculated as one of the cells in the 2 × 2 table yielded the value zero.

### Characteristics of epilepsy and seizure phenomenology

**Figure 1:**
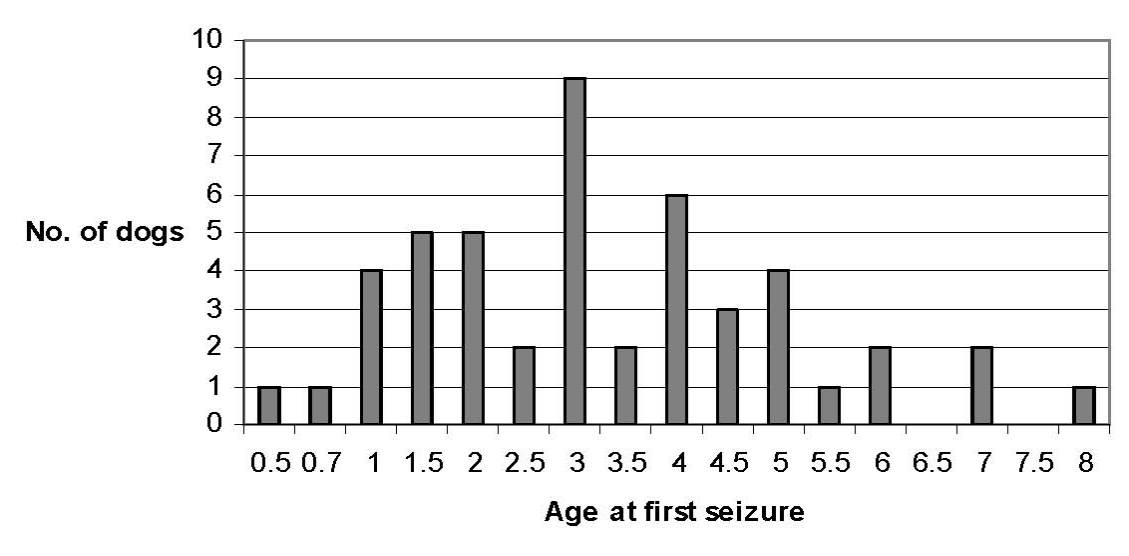
**Graphical description of age at first seizure in 49 Danish Groenendael and Tervueren dogs diagnosed with epilepsy (one missing value)**.

Mean age of debut of epilepsy was 3.3 years (range 6 months to 8 years). In 19 dogs (39%), the first seizure did not appear until after reaching the age of four years (Figure 1).

Thirty-eight dogs (78%) experienced focal seizures with or without secondary generalization. Twelve dogs experienced focal seizures alone, while 26 dogs experienced focal seizures with secondary generalization (initial focal signs followed by convulsions). Nine dogs (18%) experienced primary generalized seizures (Table 1). In two dogs (4%), seizures could not be classified. The most commonly reported focal seizure phenomenology included episodic ataxia, crawling, swaying, salivation, fearful behavior, excessive attention seeking and disorientation. The average duration of a seizure was between 30 seconds and 2.5 minutes.

In eleven dogs (22%) the owners reported that anxiety/hyperactivity/stress (e.g. working under conditions with a demand for high performance or aggressive behavior towards other dogs), could sometime act as a seizure provoking factor. These dogs would however also have seizures while resting.

In two dogs prodromes repeatedly preceded seizures. In these dogs the owners would recognize an unusual and depressed behavior that would last for several days before a forthcoming seizure episode.

None of the dogs were known to suffer from other diseases that may mimic epilepsy and all dogs appeared normal interictally and did not show any clinical signs of active intracranial disease other than seizures.

### Seizure control and mortality

In 35 dogs (71%) the general practitioner had established a clinical diagnosis of epilepsy prior to the present investigation. In thirteen dogs, 37% (13/35), the general practitioner prescribed anticonvulsive medication; eleven dogs received phenobarbital, and two dogs received phenobarbital and potassium bromide in combination. Eight dogs, 16% (8/49) had been euthanized because of failing seizure control.

In the eight dogs that had been euthanized because of epilepsy, the median time a dog lived after first seizure was 2.5 years (range 1.0–5.0 years). All eight dogs had been diagnosed with epilepsy by their general practitioner.

## Discussion

### Prevalence and study design

The present study estimated a prevalence of epilepsy of 9.5%. As very few studies have investigated the prevalence of epilepsy in canine populations, and because prevalence may vary greatly from breed to breed due to variable genetic background, data for comparisons are not available.

A prevalence of epilepsy about 1.0% has been reported from university-based populations (not breed-specific) [21,22]. Compared to this, the prevalence estimated at 9.5% in the present study appears to be high. A study by Famula and Oberbauer (2000), showed that 21% of 938 Belgian Tervueren registered in the American Kennel Club had epilepsy [18]. However, in 26 of these dogs only one seizure was observed, and thus this subpopulation did not fulfil the criteria of epilepsy used in the present study as defined by the International League Against Epilepsy (ILAE): "epilepsy is a condition characterized by recurrent epileptic seizures, numbering two or more" [20].

One might argue that owners that have a dog with epilepsy are more inclined to participate in a questionnaire survey than owners without a dog with epilepsy. If we assume that none of the dogs among the non-responders were positive, then a prevalence of 3.9% (49 dogs out of 1,248) would have been the obtained. This would imply that we have overestimated the prevalence. However, this hypothesis is counteracted by the fact that during the study, we contacted ten dog owners, who did not respond to the mailed questionnaire due to the inconvenience of filling it out and bringing it to the mailbox, even though they had a positive attitude towards the investigation. In these dogs, the investigators could confirm a diagnosis of epilepsy in all ten cases. Had they fulfilled the inclusion criteria of participating in the prevalence study by returning the mailed questionnaire, the prevalence estimate reported would have increased to 11.1%. The ten dogs provide evidence that there may be more hidden epileptic individuals in the population, whose owner did not return the mailed questionnaire due to either inconvenience or unwillingness to communicate the presence of epilepsy in their dog.

Moreover, we did not contact the owners who stated that their dogs did not have epilepsy.

Also adding to our belief that the calculated prevalence is an underestimate is the finding that in the present study, the mean age of onset of epilepsy was found to be 3.3 years. A number of dogs younger than three years may very well have been too young for having expressed the disease at the time of the investigation.

Both living and dead dogs with epilepsy were included when calculating prevalence. Excluding dead dogs would pose a risk of excluding a group of dogs with a higher prevalence of epilepsy (that might have been euthanized motivated by the severity of the epileptic condition) than the rest of the population, thus creating selection bias. Regarding recall bias, it was judged that owners would recall the rather serious signs of epilepsy even if they occurred up to 10 years back.

### Questionnaire

The mailed questionnaire was designed to detect as many possibly epilepsy-positive individuals as possible with the attendant risk of producing preliminary false-positives. A broader definition is used to identify possible cases in the study population (similar to a test with a high sensitivity but a low specificity).

Double-checking the positive responders (self-reported epilepsy) from the mailed questionnaire investigation by conducting a telephone follow-up interview emphasized the importance of validating the responses given by laymen (corresponding to a test with a high specificity ruling out the false-positive cases). Had this not been done, the study would have reported 64 epilepsy positive dogs instead of 49, the false positives leading to overestimated prevalence.

The return rate of the questionnaire was 42% which is acceptable in terms of the reliability of the calculated prevalence risk, but contains the limitation that we have described: who is willing to respond and who definitely does not want to respond.

### Case definition

In clinical studies the case definition is of uttermost importance for the reliability of the results presented. In the present study, the study design was chosen with the specific purpose to search for epilepsy positive individuals in the total population over ten years, as we wanted to determine the prevalence of epilepsy and investigate the nature of the disease in the breed in a defined time period. By nature, such a study design (including both living and dead dogs) does not allow the investigators the possibility of performing a standardized clinical evaluation of the dogs, and therefore the diagnosis of epilepsy must be based on seizure phenomenology and history as reported by the owners.

It is well known, that in epilepsy the diagnosis is to a great extend based upon the seizure history reported by the owner, and in both human and canine epileptic patients, the seizure history often holds the key to the diagnosis [20,23]. As described by the International League Against Epilepsy (ILAE), the clinical manifestation of epilepsy consists of a sudden and transitory abnormal phenomenon which may include alterations of consciousness, motor, sensory, autonomic or psychic events, perceived by the patient or an observer [20]. Seizure phenomenology and history is increasingly being used for diagnosing epilepsy in humans [19]. The validity of the diagnosis of epilepsy in the dogs presented in this study is supported by the fact that in 71% of the dogs, the general practitioner had independently of our investigation established a clinical diagnosis of epilepsy, and in the remaining dogs we did not find any indications of disease other than epilepsy when performing a thorough and extensive interview.

### Seizure characteristics and risk factors

The present study found that focal seizures, with or without secondary generalization were the most common type of seizures. Our findings are in accordance with previous studies of seizure distribution in dogs with epilepsy [4,11,22,24]. It should be stressed, that an unusually high number of individuals (25%) in this study experienced focal seizures without secondary generalization (focal seizure phenomenology not followed by convulsions), and therefore special attention should be paid to signs of focal seizure phenomenology in this breed in order to avoid that a diagnosis of epilepsy in some individuals is missed [25,26].

The Belgian shepherds are very active dogs that are easily "wand up" and in general react strongly to stressful situations involving anxiety or a high degree of excitement. In eleven dogs (22%), stressful situations would often act as an external trigger of seizures. Stress is a known trigger of seizures in humans with epilepsy and has also been reported in Labrador retrievers [27].

Unfortunately, information about the risk factors among dogs without epilepsy was not available, since the owners of these dogs were not contacted. This makes it impossible to identify risk factors for epilepsy.

A slight predilection for epilepsy has been reported for male dogs [6,9]. However, no indication of this was found in our study where around 2/3 of the cases were females. Other studies have found no difference between the sexes [8,11,12,28].

We found a highly significant risk of being euthanized because of epilepsy in intact animals as opposed to neutered dogs (*P *= 0.007). This result may indicate that a sparing effect of neutralization on seizure frequency and seizure severity exists. This could be explained by the fact that sex hormones influence seizure threshold. The convulsant/anticonvulsant effects of estrogens and progesterone, respectively, are demonstrated in women with catamenial epilepsy (seizure clustering around the time of menses) [29,30]. There is an increase in seizure frequency, immediately preceding the menstrual period, which correlates to a decrease in progesterone level, and an additional increase in seizure frequency immediately preceding the time of ovulation, correlating with a high estrogen level with no simultaneous increase in progesterone. At the end of ovulation, simultaneously with an increase in the progesterone level, the seizure frequency decreases [30]. Also, neutering may help to decrease a high level of hyperactivity/aggression/stress and thereby act as a protector against potential seizure provocation.

Finally, 5.2% of the overall study population was euthanized because of aggression showing that epilepsy is not the only grave problem experienced by the Groenendael and Tervueren variants.

## Conclusion

The present study confirms that epilepsy is a severe problem in the Groenendael and Tervueren variants in Denmark. The relatively late debut of epilepsy in the Belgian shepherd makes unattended use of epileptic dogs for breeding likely, and this contributes greatly to the ongoing increase of epileptic individuals in the breed. The severity of the disease is reflected by the fact that dogs with intractable epilepsy are euthanized.

The disease is apparently segregating in specific lines. Future studies will be performed to identify the genetic components responsible for the development of the disease.

## Authors' contributions

MB conceived of the study, participated in its design and drafted the manuscript. CHG, HGu and SLKC made a substantial contribution to acquisition of data and preparation of the manuscript. HG and LA participated in the design of the study, performed the statistical analysis and helped to draft the manuscript. MF participated in the design of the study and helped to draft the manuscript. All authors read and approved the final manuscript.
